# Jefferson Medical College

**Published:** 1844-03-09

**Authors:** H. T. Child


### CLINICAL LECTURES AND REPORTS.

### JEFFERSON MEDICAL COLLEGE.

*December* 13, 1843.

### CLINIC OF PROFESSOR MÜTTER.

(Reported by H. T. Child.)

Continued from last number.

#### CASE V.—ANCHYLOBLEPHARON AND SYMBLEPHARON.

This patient, some months since, while engaged in casting iron, was severely burned in the face and eyes, by a portion of the heated metal being accidentally thrown upon him. The left eye suffered most, and the inflammation ran so high, as not only to destroy vision, but also to occasion adhesion between the lids, (anchyloblepharon,) and between the lids and ball, (symblepharon.) These adhesions are, as you perceive, very close and strong, and it is impossible to introduce a probe between the lids and ball. The prognosis in such a case is exceedingly unfavourable; for, although we can readily divide the adhesions, yet such is the disposition to reunion, that we find it almost impossible to maintain the parts separated a sufficient length of time for cicatrization to take place. As the patient is anxious that something should be done, I will perform the usual operation of dissecting the lid from the ball, and then, in order to prevent union between the raw surfaces, shall pass a probe between them daily. It will also be well to touch the parts every day with nitrate of silver, either in solution or solid form.—Should we succeed in preventing the adhesions from re-forming, we may then introduce an artificial eye, and the deformity will at once be removed. Where the adhesions are composed of small fræna, or bands, simple division, followed by the use of the nitrate of silver, will generally relieve the deformity. The operation was then performed.

#### CASE VI.—LACERATED WOUND OF THE EAR.

In a riot among the firemen a night or two since, this man was thrown down, and while held by several persons, some one took upon himself the task of *biting off his ears.* By great good fortune, the attack was arrested after the left ear had been operated on, and before any violence could be inflicted upon the right. You perceive that what remains of the injured organ is very much inflamed, while the adjacent soft parts are in a state of erysipelas. The wound is rough and jagged, and very painful. It would be highly improper, in the present condition of the parts, to attempt any operation for the relief of the deformity occasioned by the loss of a portion of the ear, and we cannot hope to obtain either mediate or immediate union of the edges of the wound; we must therefore get down the inflammation, and then ascertain what plan will most effectually relieve the defect. To accomplish this indication, we shall order the abstraction of f℥xij. of blood from the arm, a brisk cathartic, low diet, and the warm water dressing to the wound.

When a portion of the ear has been removed by a clean cut, and the parts are not lacerated, and we are called immediately after the reception of the injury, we should always stitch on the separated parts, apply the cold or warm water dressing, and endeavour to obtain reunion. Any such attempt, when the parts are much lacerated, would prove utterly useless, as sloughing must inevitably ensue.

#### CASE VII.—CHRONIC HYDROCEPHALUS.

We have here a most distressing example of that almost indomitable disease, hydrocephalus; but as you have already, in another place, had the peculiarities of the disease pointed out, I shall barely refer to the treatment of the case before us. What is to be done for this child? The usual remedies have already been administered, but without producing any relief; the head is gradually increasing in size; the fontanelles are daily enlarging; convulsive spasms are frequent; the strabismus is perfect, and consciousness very much impaired. The prognosis is therefore very unfavourable; yet it is our duty to do something, "anceps remedium melius est quam nullam," and what promises most? The *Indian hemp in infusion.* My attention was directed to this article, as a useful remedy in hydrocephalus, by Dr. Barrow, of New York, a most excellent and experienced physician, whom I met last summer, and since that time I have used it with decided advantage in two cases. Should this fail, and the head continue to increase in size, I shall perform, as a "*dernier resort,*" paracentesis capitis, as recommended by Conquest and others, and which you saw performed at the clinic a few days since by my colleague, Prof. Pancoast. My confidence in this operation, however, is but slight; for although it has unquestionably, in several cases, proved useful, yet, in the majority, it has signally failed.

#### CASE VIII.—CONGENITAL CLUB-FOOT.

G. A., a child two years of age, exhibits the deformity known as *varus.* Both feet are involved, and the defect belongs to what I have termed, in my classification of these malformations, *varus in its third degree.* I cannot, at this time, do more than refer you to my lecture upon this subject for any information relative to the causes, complications, pathology, and treatment of similar cases. There is rarely, if ever, any difficulty in curing a child of this age by *mechanical measures alone;* but occasionally the integuments are so feeble, that the least pressure produces excoriation, or the tendon is so rigid, that a long time must elapse before its elongation can be accomplished by machinery; and when these obstacles present themselves, tenotomy will often prove useful. Before I had as much experience in the treatment of this disease as I now have, and when I was obliged to rely upon the statement of others, it was my rule to operate in all such cases as the present. I have found, however, that in *young persons,* it is as well to try a simple apparatus first, and, if this fails, we can assist it by tenotomy. (The apparatus of Dr. Mütter was then applied.)

